# Older People in Emergencies; Addressing Food Insecurity, Health Status and Quality of Life: Evaluating the “365+ Days of Care” Program

**DOI:** 10.3390/ijerph20075235

**Published:** 2023-03-23

**Authors:** Dimitrios V. Diamantis, Konstantinos Katsas, Christina Maria Kastorini, Lyndsey Mugford, Nadia Dalma, Marsellos Ramizi, Ourania Papapanagiotou, Afroditi Veloudaki, Athena Linos, Matina Kouvari

**Affiliations:** 1 Institute of Preventive Medicine Environmental and Occupational Health Prolepsis, 15121 Athens, Greece; d.diamantis@prolepsis.gr (D.V.D.); k.katsas@prolepsis.gr (K.K.);; 2 Medical School, National and Kapodistrian University of Athens, 11527 Athens, Greece; 3 Department of History of Science, Faculty of Arts and Sciences, Harvard College, Cambridge, MA 02138, USA; 4 Department of Nutrition and Dietetics, School of Health Science and Education, Harokopio University, 17676 Athens, Greece; 5 Discipline of Nutrition and Dietetics, Faculty of Health, University of Canberra, Canberra, ACT 2601, Australia; 6 Functional Foods and Nutrition Research (FFNR) Laboratory, University of Canberra, Bruce, ACT 2617, Australia

**Keywords:** older adults, natural disaster, food insecurity, food aid, process evaluation, cost-effectiveness

## Abstract

During emergencies, older adults stand among the most vulnerable, facing long-lasting food insecurity and overall health issues. The “365+ Days of Care” food aid program addressed food insecurity and poor quality of life among vulnerable older adults following a devastating wildfire in Greece. Our aim was to evaluate the program’s efficiency, using a process evaluation framework and a partial cost–utility analysis. In total, *n* = 133 wildfire-hit residents (≥65 years) received daily tailored, pre-cooked meals and/or weekly food packages. The study outcomes were assessed from baseline to 12 months later. Focus groups and interviews (*n* = 30), researcher observations, and questionnaires were used to assess the beneficiaries’ perception of the initiative. Within the 12-month follow-up period, food insecurity and malnutrition risk decreased, whereas Mediterranean diet adherence; quality of life; and physical, social, and mental health were improved (*p* < 0.05). A one-point increase in food insecurity was positively associated with improved quality of life, general health, limitation in activities, body pain, vitality, and pain/discomfort (*p*’s < 0.05), and it was marginally associated with mobility, anxiety/depression, and self-evaluated health status (*p*’s < 0.1). Quantitative and qualitative data characterized it as successful, acceptable, beneficial, and of high quality. The partial cost–utility ratio was one QALY gained per EUR 22.608. The utilization of well-designed food aid programs during emergencies can alleviate food insecurity and improve quality of life in older adults.

## 1. Introduction

Humanitarian emergencies can diminish the already weakened health status and quality of life of the vulnerable older population. Providing food to those in need after an emergency is of crucial importance. Yet most food aid programs tend to focus only on the period immediately following a disaster (critical period) [1] and provide unhealthy, energy-dense, and prepackaged meals [2]. Therefore, it is urgent to design food aid programs that effectively alleviate the physiological and mental health consequences as well as the consequent food insecurity, while respecting the beneficiaries’ dietary needs, preferences, and customs [3,4,5]. Such projects require adequate planning to be able to act immediately upon the crisis, adapt to changes, and last until the food insecurity caused by the crisis is limited. Examples of unexpected detrimental events include the COVID-19 pandemic or the recent attack in Ukraine [6], which have resulted in a significant death toll along with financial constraints, poor mental health, and food insecurity among disadvantaged groups.

The present work aimed to evaluate the “365+ Days of Care” initiative and present a report of good practice during an emergency. In 2018, during the heatwave of Attica’s coastal areas, located in central Greece, one of the wildfires ended up becoming the second deadliest in our century, with a death toll of 103 individuals, 140 hospitalizations, and nearly 4000 damaged or destroyed houses [7,8]. The resulting health consequences have overwhelmed vulnerable population groups in the region, such as older adults [9]. This program was implemented by the Prolepsis Institute and aimed to address the urgent need to address food insecurity and its impact on related physical and mental health by providing food aid tailored to older adults living in fire-stricken communities. Delivered during the disaster recovery period [1], it provided food aid to older adults and observed the impacts on food insecurity, mental health, physical health, quality of life, and dietary habits. In addition to program efficacy, this report used mixed methods and triangulation approaches to evaluate the fidelity, acceptability, impact, cost-effectiveness, and feasibility of this initiative, with a particular emphasis on process evaluation [10].

## 2. Materials and Methods

### 2.1. Setting and Participants

The “365+ Days of Care” program is a non-controlled intervention study that officially started in March 2019 and ended in April 2020. Following intense efforts to communicate the program’s availability to all eligible persons in the wildfire-hit Greek municipalities of Rafina-Pikermi and Marathonas-Nea Makri, fire-stricken residents or their close relatives/acquaintances were able to contact the Prolepsis Institute and complete an application form. Eligible participants were older adults (≥65 years) residing in the aforementioned municipalities. To ensure the absence of socioeconomic bias, place of residence, full/partial residence destruction, loss of assets, financial status, need for special nutritional care, and wildfire-attributed death or injury of close relatives were considered when selecting the participants.

Assuming that 8% of the participants would switch from food secure at baseline to food insecure at follow-up and 25% of the participants would switch from food insecure at baseline to food secure at follow-up, and after applying continuity correction, adjustment for a 10% drop-out rate, 80% power, and a two-sided level of significance of 5%, the study would require a sample size of 103 participants.

However, the program’s reach was 139 participants, who were selected from 191 applicants due to budgetary limitations, thus exceeding the required sample size and ensuring the inclusion of the highest possible number of beneficiaries. All included participants satisfied the inclusion criteria and presented at least one of the aforementioned vulnerabilities/misfortunate conditions. When someone passed away (6), another applicant took their place, resulting in a sample of 133 participants. Program retention was high, and all remaining participants completed the program. Notably, participants were not allocated to an intervention or control group, due to ethical concerns.

### 2.2. Ethical Approval

The initiative received ethical approval from the Ethical Committee of the Prolepsis Institute, carried out in accordance with the Declaration of Helsinki. All participants received information about the program’s scope, the study’s methodology, data management, and protection. All participants provided written informed consent. The program followed all applicable institutional regulations on the ethical use of human volunteers.

### 2.3. Food Aid Provision

The intervention “dose” (the quantity of intervention implemented) [10] was as follows: all beneficiaries received healthy food that aimed to cover two meals per day, corresponding to ≥60% of their daily energy needs. Beneficiaries received different types of food packages based on their current place of residence, as follows:

- For 108 beneficiaries that still resided in the fire-stricken communities, fresh, cooked lunch was provided on a daily basis along with a weekly food package with products to supplement their lunch (e.g., extra virgin olive oil) and prepare their breakfast (e.g., fruits, milk, rusks, honey, etc.). Meals and food packages were distributed at central points (military healthcare or distribution centers, public nursing homes, and local churches) or delivered to the residences of beneficiaries that were unable to reach these points or wanted to keep their inclusion in the program private.
- For the remaining 25 beneficiaries that had moved away from these areas, food packages were delivered on a weekly basis or picked up at a close central distribution point at an arranged date and time. These food packages included packaged food products to prepare their breakfast and lunch (e.g., fruits, vegetables, raw meat, fish, legumes, dairy products, bakery products, salt, extra virgin olive oil, etc.).

The program’s dietary plans and food packages were designed by a multidisciplinary expert team of dietitian nutritionists, physicians, and food technologists based on the “National Dietary Guidelines of Greece for adults aged 65 years and older” [11]. These Guidelines are designed based on the Mediterranean dietary pattern, which is tailored to older adults. Every dietary plan was further adapted to individual nutritional needs based on reported health status, preferences (e.g., vegetarian diet), religious beliefs and customs (e.g., Christian fasting), and potential allergies [12]. For management reasons, six dietary plans were created following the principles of the Mediterranean dietary pattern: general; general with no added salt; modified fiber content for moderate gastrointestinal disorders; modified fiber content for severe gastrointestinal disorders; no animal food sources; no animal food sources and no added salt. Beneficiaries received a printed weekly dietary plan along with the food packages. Prolepsis’ dieticians were in direct contact with all beneficiaries via phone for further clarifications and adjustments regarding their nutritional needs.

To ensure the hygiene and quality of the meals, strict requirements were imposed regarding the ingredients, raw materials, and preparation practices of the meals, and samples were inspected daily and regularly sent to food laboratories. Contact with the beneficiaries was carried out almost on a weekly basis, either in person or via telephone, and any arising issues or comments were promptly recorded and rectified. The project also included health promotional activities, with more details found in Appendix A.

### 2.4. Baseline Measurements

During the recruitment phase, the demographic, socioeconomic, and anthropometric characteristics; food security status; risk for malnutrition; dietary habits; quality of life; and health status of the beneficiaries were recorded. Healthcare professionals (physicians, nurses, and dieticians) were responsible for the accurate completion of the questionnaires and for resolving any queries or misunderstandings.

The food security level was measured using the U.S. Adult Food Security Survey Module 10-item questionnaire [13]. The sum of insecurity-affirming responses produces a score ranging from 0 to 10, which is categorized using a two-point scale (‘food secure adults’ (scores of 0–2) and ‘food insecure adults’ (scores of 3–10)) or a four-point scale (‘high’ (0), ‘marginal’ (1–2), ‘low’ (3–5), and ‘very low’ (6–10) food security). Malnutrition risk was evaluated using the Malnutrition Universal Screening Tool (MUST), which followed a five-step screening process to identify adults who are currently malnourished or are at risk of malnutrition [14]. The total score is classified as 0 (low risk), 1 (medium risk), and 2–6 (high risk). Adherence to the Mediterranean dietary pattern was evaluated with the use of the MedDietScore, which classifies the level of adherence to the Mediterranean dietary pattern based on a set of 11 questions, which are scored as low (score: 0–20), moderate (21–35), and high (36–55) [15].

To evaluate beneficiaries’ health status and quality of life, the 36-Item Short Form Health Survey (SF-36) and the EuroQol (EQ-5D-5L) were used. The SF-36 assesses eight health concepts and produces a score for each, along with a total score ranging from 0 (highest disability/worst possible health state) to 100 (best health state) [16,17]. The EQ-5D-5L evaluates five dimensions and corresponding scores, ranging from 1 (best health state) to 5 (worst health state) [17]. The total added score ranges from 5 to 25.

### 2.5. Follow-Up

The follow-up evaluations of the project’s effectiveness were performed by a healthcare professional at 6 (110 beneficiaries; 82.7% participation rate) and 12 months (133 beneficiaries; 100% participation rate) after the recruitment phase. The 6-month evaluation was performed in person, while the 12-month evaluation occurred via phone interview due to COVID-19 restrictions.

In order to examine whether such a program was agreeable and feasible for replication as a best practice, the participants completed questionnaires assessing program satisfaction and acceptance at 12 months (*n* = 132, 99.2% participation rate). This survey assessed a number of acceptability metrics, including the quality, taste, variety, and frequency of meals and packaged foods; communication with the program’s organizers; assessment of dietary improvements; suggestions for improvement; and general satisfaction.

### 2.6. Statistical Analysis

Quantitative variables are presented as mean ± standard deviation (SD) for those with a normal distribution and as median (interquartile range, IQR) for non-normally distributed variables. *t*-tests were performed to compare the means of normally distributed quantitative variables between two groups, and Mann–Whitney U tests were used for variables with non-normal distributions. For three or more groups, ANOVA tests were utilized for normally distributed variables, and Kruskal–Wallis H tests were used for variables with non-normal distributions. Differences in a variable between baseline and each follow-up (pre–post analysis) were investigated using paired *t*-tests for normally distributed quantitative variables and Wilcoxon matched-pairs signed-rank tests for non-normally distributed variables. Exact symmetry tests (for KxK tables) and McNemar tests (for 2 × 2 tables) were utilized for pre–post analyses regarding categorical variables. We used multi-adjusted logistic regression models to investigate the likelihood of improvement in quality of life (SF-36 health concepts and EQ-5D health dimensions baseline and 2nd follow-up scores) depending on the increase in food insecurity score. Differences in food insecurity scores were calculated as the negative difference between food insecurity scores at the 2nd follow-up minus the score at baseline (a one-point increase indicated improvement in food security). All independent variables were checked for collinearity. The level of significance was defined as α = 0.05. All analyses were performed using SPSS statistics 26.

### 2.7. Cost-Effectiveness Analysis

A partial cost–utility analysis was performed, due to the lack of a control group, in which the cost of the meal provision was compared with the total quality-adjusted life years (QALYs) gained by the intervention [18]. The EQ-5D-5L health states were utilized to calculate the weighted health state indexes (index values), by applying the value sets for the United Kingdom population and, thus, calculating the QALYs gained [19]. Index values range from −0.288 to 1, with values equal to 0 representing death, values equal to 1 indicating a perfect health state, and negative values indicating a health status worse than death. As the project lasted for one whole year, the derived index values, representing the utility score (total utility index), are equal to the QALYs gained during this timeframe.

Regarding the projects’ cost, a detailed report of the implementation expenses was organized, which included expenses related to food and meal supplies, packaging costs, transportation, and operating expenses, researchers and related staff wages, communication- and managerial-related expenses, questionnaire development and distribution, and focus group implementation. The overall project cost was EUR 378,573.09.

### 2.8. Qualitative Assessment

Beneficiaries were selected at random to be interviewed eight months after program initiation (during November 2019) to evaluate it in terms of describing their incentives to participate and remain in the program, assessing the implementation methods, and proposing potential modifications for further consideration. The qualitative study was comprised of (i) three 45–60-min focus groups with 8 beneficiaries each receiving daily meals and (ii) six 30-min personal interviews with beneficiaries receiving weekly food packages (total *n* = 30). Focus groups and personal interviews were conducted until saturation was reached.

A discussion guide was developed by the multidisciplinary research team (nutritionist, psychologist, sociologist, and public health experts). The guide aimed to evaluate the beneficiaries’ initial motivations, the program’s benefits and drawbacks, communication with support workers, satisfaction with the implementation procedures, and proposals for program optimization.

Two researchers were present at all focus groups/interviews. The senior moderator posed the questions and monitored the conversation, while the assistant moderator kept notes of the responses and ensured the conversation’s recording. Transcripts were analyzed independently by two researchers following the thematic analysis approach. To enhance the study’s validity, recurrent themes and subthemes were compared, revised, and refined between the two researchers. The surrounding discussions from which indicative themes and subthemes arose were evaluated to ensure proper interpretation.

### 2.9. Observations

To create a complete picture of program effectiveness, following rigorous training in observational methods by experts in qualitative analysis, researchers conducted regular observations of the meal preparation and provision on site and observed the beneficiaries, meal preparation staff, and volunteers without interfering with the process of meal distribution. A total of 4 observation sessions were performed each month (weekly basis). Findings were collected in a notebook, and then a report was compiled and presented to the program coordinators. However, observations were not only available from researchers but also from volunteers, community members, and caretakers. They were asked to observe and report any implementation issues to the researchers on site as soon as they noticed them or to the project coordinator by phone. Their input was then recorded and presented to the project coordinator.

### 2.10. Triangulation

By combining the stakeholders’ observations, the qualitative data, and the quantitative data on effectiveness, method triangulation was achieved [20]. Triangulation can enhance the study’s credibility by combining multiple sources of data and perspectives to provide a comprehensive understanding [21,22] and ensure a better picture of the project’s effectiveness and acceptability and beneficiaries’ experience. It should be noted that each researcher performed only one of the tasks, based on their expertise: (a) analyzed the quantitative data, (b) collected the qualitative data, or (c) performed the observation sessions.

## 3. Results

### 3.1. Sociodemographic Characteristics of Beneficiaries

Beneficiaries had a mean age of 72 ± 9 years, 37.6% were males, and 58.7% were married (Table 1). Overall, 62.1% had lost their house due to the fire, 50% had lost other assets, and 8.3% had lost a first-degree relative in the wildfire events. Safe running water at home was unavailable for 21.1% of the beneficiaries.

### 3.2. Program’s Effectiveness

The impact of the “365+ Days of Care” program on food insecurity, malnutrition risk, BMI, level of adherence to the Mediterranean diet, quality of life, and health status from baseline to 2nd follow-up is reflected in Table 2. Significantly lower scores in food insecurity (−2.2 ± 3.4 difference) and malnutrition (−0.17 ± 0.83 difference) were observed after one year, with only 34.8% of adults being food insecure compared to 61.8% at baseline (*p*’s < 0.05). Adherence to the Mediterranean diet was improved (score increment by 2.7 ± 4.2), with 27.1% of beneficiaries reporting high adherence, compared with 11.5% at baseline (*p* < 0.001).

All quantitative variables are presented as mean ± standard deviation, except BMI (because it is not normally distributed), which is presented as median (interquartile range). We used paired *t*-tests for normally distributed quantitative variables and Wilcoxon matched-pairs signed-rank tests for non-normally distributed variables (BMI). Exact symmetry tests (for KxK tables) and McNemar tests (for 2 × 2 tables) were utilized for pre–post analyses regarding categorical variables.

The total quality of life scores, as well as all subscales of the SF-36 and EQ-5D questionnaires, indicated significant improvement (*p* < 0.05). The only exception was self-care (EQ-5D). SF-36 improved by 10.5 ± 17.2 and EQ-5D declined by 2.0 ± 3.7 (*p*’s < 0.001), demonstrating an improvement in quality of life. Beneficiaries reported augmentations in general, physical, emotional, and mental health; social life; and vitality but also less limitation in activities and body pain (SF-36 health concepts) (*p*’s < 0.05). Alleviation in pain/discomfort, anxiety/depression, and mobility issues was also evident (EQ-5D health dimensions) (*p*’s < 0.05). The aforementioned subscale’s mean scores can be found in Table 2.

In Table 3, the results of muti-adjusted logistic regression models investigating the association between food insecurity alleviation and the likelihood of experiencing improvement in quality of life (via improvement in SF-36 or EQ-5D scores). A one-point increase in food insecurity score was associated with evident improvement in total quality of life (SF-36: OR = 1.29; 95%CI [1.02–1.64] and EQ-5D: OR = 1.30; 95%CI [1.06–1.61]), general health (OR = 1.35; 95%CI [1.13–1.63]), body pain (OR = 1.18; 95%CI [1.01–1.38]), and vitality (OR = 1.24; 95%CI [1.05–1.47]) and reduction in limitations in everyday activities (OR = 1.22; 95%CI [1.04–1.43]) and pain/discomfort (OR = 1.22; 95%CI [1.04–1.43]). Food insecurity alleviation was also marginally associated with a reduction in anxiety/depression (OR = 1.17; 95%CI [1.00–1.36]) and improvements in mobility (OR = 1.15; 95%CI [0.99–1.34]) and self-evaluated health status (OR = 1.15; 95%CI [0.99–1.35]).

### 3.3. Reported Acceptability and Satisfaction of Program’s Beneficiaries

Almost all beneficiaries reported that they were satisfied with the quantity (94.7%), quality (90.9%), and taste (90.9%) of the provided products and meals. The meals and food packages were delivered on time, and no issues were presented regarding communication with the Prolepsis team. In addition, due to their participation in the program, beneficiaries tended to eat healthier overall (97%); in particular, they reported an increase in the consumption of fruits (97%), vegetables (97%), dairy products (98.5%), fish (96.2%), whole grain products (93.2%), and pulses (84.1%). Beyond these, almost all beneficiaries felt cared for and less lonely, with the program facilitating their quality of living and supporting them financially (by covering part of the cost of buying food). The overall satisfaction score was 9.5 ± 0.7 out of 10, with a range of 7–10. However, a small number of beneficiaries reported that they would prefer for the program to cover their daily home utility needs (e.g., napkins, detergent) (*n* = 3) and provide physiological support (*n* = 1).

### 3.4. Economic Evaluation and QALYs

**Figure 1:**
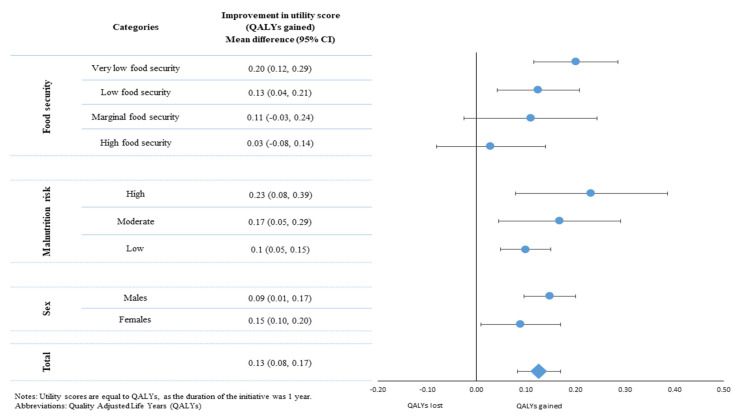
Mean differences in utility values and QALYs gained by the total sample as well as according to sex, food insecurity status, and malnutrition risk classification.

The mean difference in the utility index was 0.13 ± 0.26 (*p* = 0.02), which translates to an average of 0.13 QALYs gained per person, yearly (Figure 1). Overall, participants gained 16.8 QALYs from their participation. The cost–utility ratio was computed as one QALY gained per person at the cost of EUR 22.608 (equal to USD 25,690 or GBP 20.038 at the 2020 exchange rate [23]). A proportional trend between improvements in QALYs with higher food insecurity was observed, with the high food security group reporting a non-significant improvement of +0.03 QALYs (95%CI: −0.08, 0.14) and the very low food security group reporting an improvement of +0.20 QALYs (95%CI: −0.12, 0.29).

### 3.5. Qualitative Findings

#### 3.5.1. Focus Groups and Interviews

Qualitative analysis revealed the following three main themes regarding the beneficiaries’ characterization of the program: necessary for their health and needs, proper social initiative, and high-quality offered meals tailored to their dietary needs. Participants stressed the importance of the program not only as an urgent supportive measure right after the wildfire but as a general supportive measure for older adults facing further social difficulties as well as having poor health. A description of each theme, as well as representative quotes, can be found in Table 4. Overall, no particular issues with the program’s procedures were reported through the focus groups or interviews.

#### 3.5.2. Observations

The researchers/observers reported the following as their main observations, based on their weekly observation sessions. First, the act of daily meal provision proved effective at reassuring the beneficiaries that someone was looking after their health and provided an opportunity for social interaction. Beneficiaries were accustomed to the volunteers, and the volunteer’s friendly attitude evidently boosted the beneficiaries’ morale. This was also confirmed by the on-site volunteers and meal providers, through their telephone communication with the institute and discussions with the researchers responsible for the observation sessions. Secondly, the distribution points and the telephone line proved to be an efficient way to communicate any dissatisfaction or need for dietary modification and efficiently change the corresponding meal. The process of dietary modification was as follows: the beneficiary informed the on-site volunteers, who later informed the Institute’s dieticians, or the beneficiary was directly linked with a dietician via telephone. The dietician then considered the necessary adjustments or changes to a different meal type and informed the on-site volunteers and the meal preparation staff of specific modifications. This process lasted for about a day and resulted in a tailored meal the following day. Finally, caretakers, community members, and researchers all reported that meal modifications further improved the beneficiaries’ morale and limited the likelihood of a beneficiary leaving the program.

### 3.6. Program Fidelity and Adaptability

Program fidelity assesses the degree to which an intervention is delivered according to plan [10]. Firstly, while participant communication was always included for morale and feedback purposes, the communication frequency (nearly weekly) increased in response to beneficiary requests and feedback. Dietary plans were often modified following beneficiary feedback and the ingredients’ seasonality. Finally, the emergence of the COVID-19 pandemic forced the program to adopt new methods to protect beneficiaries’ safety in March–April 2020. In these months, distribution adopted new hygiene protocols, and beneficiaries who felt unsafe could opt into at-home meal delivery. Otherwise, the intervention was delivered as planned.

## 4. Discussion

The “365+ Days of Care” initiative, overall, proved to be effective at reducing the long-lasting food insecurity, malnutrition risk, and disability-related health consequences among older adults living in a fire-stricken community. Moreover, this initiative favorably affected dietary habits, while, at the same time, positively affecting mental health, social activities, quality of life, and the perceived health status of the beneficiaries. Food insecurity alleviation was positively associated with improvements in quality of life, indicators of body health (i.e., less limitation in activities, body pain, depression, and more vitality), and, potentially, mental health. Quantitative, qualitative, and stakeholder assessments all indicated that the initiative was viewed as successful, acceptable, beneficial, and of high quality. The program was able to offer a substantial improvement in QALYs for a reasonable cost. This intervention, which provided high-quality, tailor-made meals to vulnerable older adults, can be used as an example of good practice not only for reducing food insecurity but also for ameliorating general, mental, and social health.

To the best of our knowledge, this is the first post-disaster food aid initiative that has indicated an overall improvement in health in older individuals during the early recovery and year-long recovery periods. In addition, the program seemed to limit long-lasting food insecurity and malnutrition risk. Long-lasting, post-disaster negative general and mental health outcomes [3,24,25] can predict long-lasting, persistent food insecurity [3]. Furthermore, in vulnerable groups, mental health, post-traumatic stress disorder, and even food insecurity can take longer to improve, which translates into a substantial disease burden [3,4,5]. Similar initiatives are scarce in the literature, with the use of high-quality, pre-prepared snacks, but these have limited timeframes of action and effectiveness indicators [26].

This initiative is unique from other food aid programs because it prioritizes flexibility, communication, connection, and personalization in addition to food delivery, efficient fund allocation, and the consideration of beneficiaries’ satisfaction. Humanitarian aid initiatives must be quick, responsive, and designed in an effective way to account for these unexpected events and aim for long-term positive outcomes [5,27,28]. The program’s fidelity and adaptability even proved adequate during unpredictable conditions, such as the COVID-19 quarantine, as rigorous processes and changes relevant to the beneficiaries’ reach proved to ameliorate the negative effects of quarantine on their mental health and quality of life [29,30]. This feature, including opportunities for feedback, also allows the program to be tailored to the needs of specific contexts and communities, as it provides a dynamic framework rather than a rigid one-size-fits-all approach.

The partial cost–utility analysis provided evidence of substantial benefit in QALYs for a reasonable cost of USD 25,690 (GBP 20,038) per additional QALY. The incremental cost-effectiveness ratio, developed by the NICE, states that an intervention costing less than GBP 20,000 per additional QALY is considered cost-effective, while willingness to pay for additional health benefits in the US can even be USD 100,000–150,000 [31,32,33]. However, these rates are based on full-on economic evaluations and are often related to drug treatments. If we considered the lack of food aid provision following a disaster, our intervention would have potentially seemed just as cost-effective; however, in reality, food aid is most often provided following a disaster without accounting for cost-effectiveness. Moreover, the provision of ready-to-eat, military-like, pre-packaged meals, without taking into account the needs and preferences of vulnerable populations, can prove to have such limited effectiveness that it overcomes its low-cost advantage [27,34].

### Limitations

The initiative’s humanitarian scope and ethical principles did not allow for the design of a controlled interventional study, which could have increased our results’ credibility. Consequently, a full economic evaluation was infeasible. Another limitation is the lack of formal provision of mental and general support beyond the continuous communication conducted with all beneficiaries [35,36,37]. However, the initiative has proven effective at tackling poor mental health without even accounting for the expected surge in anxiety, sadness, and poor mental health attributed to the COVID-19 pandemic during the final re-audit [29]. A potential source of response bias was the alteration of the face-to-face final follow-up to a telephone re-audit [38], which was minimized by comparing the 6-month and 12-month effectiveness and observing a positive trend in improvement in most factors. Finally, the index value, set for the UK population, was utilized since relevant values for the Greek population were unavailable.

## 5. Conclusions

Following an emergency, older adults can experience long-lasting food insecurity and poor quality of life. Providing food aid that values their needs, customs, and preferences can effectively reduce the consequent health burden and positively impact their quality of life and dietary habits. Future initiatives should value the beneficiaries’ needs, preferences, and customs; closely monitor the quality and safety of the provided food; and adapt their processes based on beneficiaries’ satisfaction and needs. Initiatives should be designed with quick adaptation to changes in needs or other disasters and have a close connection with the beneficiaries constantly. Post-disaster, long-term health consequences and food insecurity in vulnerable populations should not be underestimated and must be effectively targeted through tailored food aid initiatives.

## Figures and Tables

**Table 1:** Baseline sociodemographic characteristics (*n* = 133).

Age, Years *	72 ± 9
Men, %	37.6
Marital status, %	
*Living alone*	
*Single*	6.8
*Widowed*	24.8
*Divorced*	9.8
*Cohabitation*	
*Married*	58.7
Nationality, % Greek	96.2
Insurance, % no	11.4
Electricity at home, % no	3.0
Safe drinking water at home, % no	21.1
House damage, %	
*Completely destroyed*	62.1
*Severe damage*	34.1
*No damage*	3.8
Loss of other assets, % yes	50
Loss of a first-degree relative, % yes	8.3

* Age is presented as mean ± standard deviation.

**Table 2:** Food insecurity, risk for malnutrition, BMI, adherence to the Mediterranean diet, quality of life, and health status at baseline and 2nd follow-up.

	Baseline (*n* = 133)	2nd Follow-Up (*n* = 133)	Difference
Food insecurity status			
*Food insecure, %*	61.8	34.8	−27 **
*Food insecurity score*	4.4 ± 3.3	2.2 ± 2.7	−2.2 ± 3.4 **
Risk for malnutrition, %			
*Low*	78.3	89.1	+10.8% *
*Medium*	11.7	4.2	−7.5% *
*High*	10	6.7	−3.3% *
MUST score (range: 0–6)	0.34 ± 0.74	0.18 ± 0.53 *	−0.17 ± 0.83 *
Weight status, %			
*Underweight*	0	0.8	+0.8%
*Normal*	24	25.6	+1.6%
*Overweight*	44	43.2	−0.8%
*Obese*	32	30.4	−1.6%
BMI, kg/m^2^	27.7 (25.2–32)	27.7 (24.9–30.9)	0.0
Mediterranean diet			
Level of adherence to Mediterranean diet, %			
*Low*	0.0	0.0	0.0% **
*Medium*	88.5	72.9	−15.6% **
*High*	11.5	27.1	+15.6% **
MedDietScore (range: 0–55)	31.1 ± 3.8	33.8 ± 4.2	+2.7 ± 4.2 **
SF-36 health status questionnaire			
*Total score*	41.7 ± 19.5	52.3 ± 20.2	+10.5 ± 17.2 **
*General health*	40.1 ± 21.8	45.4 ± 20.6	+5.4 ± 20.3 *
*Limitations of activities*	49.4 ± 32.8	54.4 ± 32.7	+5 ± 35 *
*Physical health*	26.4 ± 40.2	50.4 ± 46.5	+24.4 ± 53.6 **
*Emotional health*	27.5 ± 39.6	51.7 ± 41.2	+24.2 ± 54.1 **
*Social activities*	40.3 ± 33.3	56.7 ± 30.2	+16.3 ± 40 **
*Body Pain*	40.1 ± 31.9	55 ± 34.1	+15 ± 32.1 **
*Vitality*	36.5 ± 23.9	47 ± 22.3	+10.5 ± 22.7 **
*Mental health*	44.8 ± 21.5	56.3 ± 21.5	+11.5 ± 21.5 **
EQ-5D QOL			
*Total score*	13.9 ± 4.8	11.9 ± 4.8	−2.0 ± 3.7 **
*Mobility*	2.78 ± 1.35	2.44 ± 14	−0.34 ± 1.1 **
*Self-care*	1.76 ± 1.26	1.68 ± 1.22	−0.08 ± 1.03
*Usual activities*	2.79 ± 1.31	2.33 ± 1.32	−0.46 ± 1.27 **
*Pain/Discomfort*	2.98 ± 1.26	2.53 ± 1.23	−0.45 ± 1.16 **
*Anxiety/Depression*	3.59 ± 1.18	2.9 ± 1.15	−0.68 ± 1.26 **
Self-evaluated health status	53.1 ± 20.0	59.1 ± 21.5	+6 ± 19.3 **

** *p* < 0.001; * *p* < 0.05. Abbreviations: QOL, quality of life; SF-36, 36-item short-form health survey.

**Table 3:** Multi-adjusted logistic regression with the independent variable as the change in food insecurity score (improvement of food security) * between baseline and the 12-month follow-up and the dependent variable being the respective quality of life parameters.

	OR (95% CI)	*p*-Value
Improvement of participants’ health status, defined through the SF-36, yes/no		
*Overall health*	1.29 (1.02, 1.64)	0.036
*General health*	1.35 (1.13, 1.63)	0.001
*Limitations of activities*	1.22 (1.04, 1.43)	0.013
*Physical health*	1.00 (0.87, 1.15)	0.995
*Emotional health*	1.07 (0.92, 1.23)	0.388
*Social activities*	1.06 (0.91, 1.22)	0.461
*Body pain*	1.18 (1.01, 1.38)	0.033
*Vitality*	1.24 (1.05, 1.47)	0.013
*Mental health*	1.04 (0.88, 1.22)	0.647
Improvement of participants’ health status, defined through the EQ-5D QOL, yes/no		
*Overall health*	1.30 (1.06, 1.61)	0.014
*Mobility*	1.15 (0.99, 1.34)	0.063
*Self-care*	1.18 (0.94, 1.48)	0.143
*Usual activities*	1.06 (0.92, 1.22)	0.408
*Pain/Discomfort*	1.22 (1.04, 1.43)	0.014
*Anxiety/Depression*	1.17 (1.00, 1.36)	0.051
*Self-evaluated health status*	1.15 (0.99, 1.35)	0.073

* Change in food insecurity score = −(food insecurity score at follow-up − baseline food insecurity score); higher values indicate better improvement in food security. Abbreviations. QOL, quality of life; OR, odds ratio; 95% CI, 95% confidence interval; SF-36, 36-item short-form health survey, EQ-5D, EuroQol EQ-5D-5L. All models have been adjusted for age, sex, total or partial household damage, and the existence of a cohabitant in their household (living alone or being married).

**Table 4:** Qualitative findings, taken from combining focus groups and personal interview findings, assessing the beneficiaries’ perceptions of the program implementation and effectiveness.

Main Themes	Description	Representative Selected Quotations
Immediate needs of beneficiaries attributable to their emergency.	Beneficiaries emphasized problems related to depression and hopelessness, poverty, and poor food infrastructure in temporary accommodations; no kitchens or fridges, thus allowing no dietary flexibility regarding health needs.	*“There is a need for this program. It’s not only a financial need. It’s also psychological.” (73 y., male, daily meal recipient)* *“What you do for us is very important. Both practically and psychologically, because there are so many difficulties, and we are not young, to have all our lives ahead of us.” (81 y., male, weekly package recipient)*
Proper social initiative.	Beneficiaries stated that sometimes aid programs create social stigma for participants that implies pity, exclusion, or helplessness. Participants did not indicate any such feelings, and instead praised the program’s supportive nature. They felt supported, respected, loved, and cared for.	*“The program is a support with love. It offers love.” (71 y., female, daily meal recipient)* *“I have not felt offended. I felt only love and support.” (75 y. female, weekly package recipient)* *“The fact that you ask for our opinion is essential.” (69 y., male, weekly package recipient)*
High-quality food aid provisions tailored to their nutritional and health needs.	Participants highlighted that many of the program’s unique components were essential to their satisfaction and experience. These elements included meal quality and freshness, special menus based on religious and health needs, daily food monitoring by the organization responsible for the program, and frequent communication with those working in the program in order to provide feedback on the implementation methods as well as the actual meals.	*“My husband would not have had such a good blood-test, if he were not eating the special meal offered to him. Because we live in camps, we can’t cook, we don’t have a kitchen, so we eat what they give us. And what you give to us has quality and it is based on our special health needs.” (70 y., female, daily meal recipient)* *“The whole program has quality. No expired products or close to expiration. What you give us in packages is excellent. We would buy them ourselves.” (83, male, daily meal recipient)* *“I fast (Christian fasting) and you bring me the menu for those who fast. I appreciate it very much.” (87, female, daily meal recipient)* *“Checks are necessary and we appreciate it because we know we are eating something good. Monitored.” (71 y., female, daily meal recipient)* *“We see great interest and we have meaningful communication with employees. It’s not that they just throw something (meal) at us and leave.” (73 y., male, daily meal recipient)*

## Data Availability

The data presented in this study are available upon request from the corresponding author.

## Appendix A. Supplementary Methods

In addition to meal and food package provision, the program included health promotion activities for the wider community, targeting senior citizens. Two information days took place in the fire-stricken communities. The first one, entitled “Healthy nutrition in the third age” took place in September 2019 and targeted older people in the community. The second, in November 2019, was entitled “Food hygiene and safety”, and was aimed at volunteer cooks who were responsible for preparing daily meals for the beneficiaries. Free medical examinations were implemented for all beneficiaries, 65 of whom participated in further physician consultation, either at a hospital or through a mobile medical unit for beneficiaries with mobility issues. Finally, a celebration event took place on New Year’s Eve, with student volunteers preparing gifts for the beneficiaries, fundraising for the initiative, and participating in an event with traditional dances, music, and intergenerational discussions.
